# Multiprofessional prescribing and continuity of pre-exposure prophylaxis use: The role of pharmacy and nursing in strengthening access within a universal public health system

**DOI:** 10.1016/j.rcsop.2026.100834

**Published:** 2026-08-28

**Authors:** Francisco Álisson Paula de França, Ana Luiza Alves dos Santos, Gabriel Gonçalves Okamoto, Maria Christina dos Santos Verdam, Débora Santos Lula Barros, Noemia Urruth Leão Tavares, Rodrigo Fonseca Lima, Rafael Santos Santana

**Affiliations:** a Laboratory of Pharmaceutical Evidence and Studies (LEFAR), Faculty of Health Sciences, University of Brasília, Brasília, DF, Brazil; b State Health Department of the Federal District, Brasília, DF, Brazil

**Keywords:** PrEP, Pharmacists, Nursing, Health equity, Task shifting

## Abstract

**Objective:**

To analyze the association between prescriber professional category and continuity of oral pre-exposure prophylaxis (PrEP) use, identifying the contribution of nursing and pharmacy to strengthening access and retention among vulnerable populations.

**Methods:**

Cross-sectional study using secondary PrEP monitoring data from Brasília, the Federal District of Brazil, covering new users registered from October 2024 to December 2025. The final sample comprised 2317 individuals with at least one oral PrEP dispensation. Pharmacist prescribing began after training in August–October 2024. Descriptive statistics, Pearson's chi-square test, and multivariate logistic regression were applied (*p* < 0.05).

**Results:**

Pharmacist care was concentrated in primary care (91.0%; *n* = 389) and exclusively in the public sector (100.0%; *n* = 427), while physicians worked predominantly in the private sector (63.1%; *n* = 779) and nurses in specialized services (73.3%; *n* = 480). Nursing and pharmacy served proportionally more Black and mixed-race users (60.2% and 57.0%, respectively) than medicine (45.0%). Nursing showed greater reach among incarcerated people, substance users, and sex workers (3.7%, 4.3%, and 5.0%, respectively). Twelve-month continuity was higher among pharmacist-followed users (97.9%; *n* = 418) than nurses (80.6%; *n* = 528) and physicians (72.4%; *n* = 894). Multivariate regression confirmed professional category as an independent predictor of continuity, with higher adjusted Odds Ratios (aOR) and 95% confidence intervals (95% CI) for pharmacist-followed (aOR = 4.25; 95%CI 2.10–8.50; *p* < 0.001) and nurse-followed users (aOR = 1.65; 95%CI 1.30–2.10; *p* = 0.002) than the physician-led model.

**Conclusion:**

Integration of pharmacist prescribing and the leading role of nursing within the public network may broaden reach among historically vulnerable populations and increase initial continuity, supporting decentralization as a strategy to reduce inequities in access to combination Human Immunodeficiency Virus (HIV) prevention technologies.

## 1 Introduction

Human Immunodeficiency Virus (HIV) infection remains a public health priority worldwide. Without adequate treatment, HIV progresses to Acquired Immunodeficiency Syndrome (AIDS), a stage marked by severe immune compromise.^1^ In response to the epidemic, pre-exposure prophylaxis (PrEP) has become established as a highly effective biomedical strategy for preventing infection, capable of reducing the risk of viral acquisition by up to 99% when used appropriately.^2^

In Brazil, PrEP is provided through the Brazilian Unified Health System (SUS), which guarantees universal access to healthcare. Initially, eligibility was prioritized for key populations and groups at greater vulnerability, including gay men and other men who have sex with men (MSM), transgender people, travestis (a distinct gender identity in the Latin American context, not synonymous with transgender women), and sex workers. Currently, PrEP is recommended for any person at substantial risk of HIV acquisition, regardless of population group.1., 3 Despite free provision through the SUS, structural and social barriers such as stigma, discrimination, and service centralization continue to limit equitable access. Epidemiological data indicate that transgender and travesti populations, for example, remain underrepresented among PrEP users, highlighting the need for innovative strategies to reach the most vulnerable.^4^, ^5^, ^6^

In this context, decentralization of care through task-sharing is globally recognized as a tool for expanding health coverage.^7^, 8. The inclusion of non-physician professionals, such as nurses and pharmacists, in PrEP prescribing and management has demonstrated safety and effectiveness across various health systems, facilitating the reach of actions within primary health care.9., 10

Oral PrEP was incorporated into Brazil's SUS in December 2017, with nationwide implementation completed over the following years, establishing free access as a cornerstone of the country's combination HIV prevention strategy.^1^ Outside the public network, PrEP can also be obtained through private clinics and pharmacies, where consultation and medication costs are borne directly by patients or through private health insurance.^18^ To expand access within the public health system, the Federal Pharmacy Council (Conselho Federal de Farmácia, CFF), in alignment with the Ministry of Health, authorized pharmacists to prescribe PrEP and post-exposure prophylaxis (PEP).^11^, 12., 13. This policy positioned Brazil among the first countries in Latin America to permit pharmacist prescribing of PrEP and PEP within a public health system. By regulation, this authorization is restricted to the SUS and does not extend to the private sector.

Integration of pharmacist prescribing aims not only to increase points of access but also to improve the quality of care, promoting health and greater continuity of prophylaxis use within services.^14^, 15. However, comparative evidence remains scarce between the profile of users seen by pharmacists and those followed by physicians and nurses, particularly regarding the ability to reach historically marginalized groups and promote equity.

Given the recent implementation and training of pharmacists for PrEP prescribing within the public network of Brasília in 2024, comparative analysis between prescribing professional categories constitutes a necessary step to support decision-making on the expansion of this policy.16., 17 Analysis of access according to sociodemographic characteristics, vulnerability, and continuity of use, operationalized through an indirect retention indicator based on dispensation regularity, allows identification of differentiated patterns between professional categories and their implications for equity in access to combination prevention technologies.^18^, ^19^, ^20^

Therefore, this study aimed to analyze the association between prescriber professional category and continuity of oral PrEP use, identifying the contribution of nursing and pharmacy to strengthening access and retention of vulnerable populations.

## 2 Methods

This is a cross-sectional study using publicly available secondary data from HIV pre-exposure prophylaxis (PrEP) monitoring in Brasília, the Federal District of Brazil. The setting comprised health services affiliated with the Federal District Health Department (Secretaria de Saúde do Distrito Federal, SES-DF). This initiative, linked to the Pharmaceutical Services Protocols Program of the Federal District's SUS (Profarma-SUS) and aimed at developing the clinical competencies of pharmacists working across different levels of the health care network, may have supported the introduction of pharmacist prescribing into the decentralization and task-sharing pathways analyzed in this study.

Data collection took place between January and February 2026, covering registrations of new users made between October 2024 and December 2025. This specific timeframe was established to coincide with the period following the introduction of pharmacist prescribing into the health network, aiming to obtain a comparison between professional categories operating within the same interval and mitigating potential distortions arising from historical registration patterns in prior years.

The study population comprised newly registered users who began oral PrEP use in public health services within the Federal District exclusively within the analyzed interval. Duplicate records and those linked to professional categories with low numerical representativeness, such as exchange-program physicians, were excluded from the sample.

For operationalization of the study, prescriber professional category (physician, nurse, or pharmacist) was defined as the main independent variable. Dependent variables and indicators were organized into five analytical dimensions: (1) service network, dispensations, and users; (2) user profile; (3) discontinuity; (4) prescribing in private services; and (5) origin of care. The indicators used within each dimension followed a model adapted from the national PrEP clinical monitoring report^1^ (Table 1). Within the user-profile dimension, a vulnerability analysis further assessed the distribution of programmatic and social vulnerability markers, including experiencing homelessness, incarceration, alcohol misuse, psychoactive substance use, and sex work, across prescribing professional categories (Table 4).

**Table 1:** Variables analyzed in the study.

**Dimension**	**Indicators**
**Service network, dispensations, and users**	Number of selected services that dispensed oral PrEP within the Federal District
	Number of oral PrEP dispensations
	Number of users with at least one oral PrEP dispensation during the analyzed period (Oct 2024–Dec 2025)
	Number of users on oral PrEP in December 2025
	Number of people in oral PrEP discontinuation in December 2025
**User profile**	Total number of people on oral PrEP
	Distribution of oral PrEP users by population
	Distribution of oral PrEP users by age group
	Distribution of oral PrEP users by education level
	Distribution of oral PrEP users by self-declared race/skin color
**Discontinuity**	Number of people who received at least one oral PrEP dispensation during the analyzed period and who, in December 2025, were in discontinuation
	Proportion of oral PrEP users in discontinuation, by population
**Prescribing in private health services**	Proportion of users initiating oral PrEP whose care was provided in the private sector
**Origin of care**	Proportion of oral PrEP encounters, by service type, by month of care

Continuity of oral PrEP use (yes/no), derived from the discontinuity dimension, was used as the dependent variable in the multivariate regression analysis (Table 5).

Among the indicators analyzed, treatment adherence was operationalized through the rate of return for a new PrEP dispensation. For assessment of immediate adherence, dispensation was considered valid within the prescribed period plus a 40% tolerance, classifying return as timely (within 42 days for 30-day dispensations; within 84 days for 60-day dispensations; and within 168 days for 120-day dispensations). In parallel, retention in clinical follow-up was assessed, measured by maintenance of PrEP use over a 12-month horizon. For users with follow-up time equal to or greater than 12 months, timely-adherence and retention criteria were applied cumulatively, whereas for those with less than 12 months of follow-up, timeliness of dispensation according to the prescribed period was considered. This design allowed an integrated analysis of user engagement and continuity of care according to prescriber professional category. This tolerance threshold follows the operational definition adopted in the Brazilian Ministry of Health's national PrEP and PEP monitoring report.^1^

Data analysis used Microsoft Excel® and RStudio® software. Descriptive analysis was performed using absolute and relative frequencies. To verify statistical association between professional category and user profile, Pearson's chi-square test was applied, with a significance level of 5% (*p* < 0.05). Additionally, to identify independent factors associated with continuity of PrEP use, multivariate binary logistic regression analysis was performed. Variables showing statistical significance in bivariate analysis (*p* < 0.05) were included in the model to adjust for potential confounding factors. The dependent variable was defined as continuity of prophylaxis use (Yes = 1; No = 0). Results were expressed as adjusted Odds Ratios (aOR), with respective 95% confidence intervals (95% CI). The analytical approach sought to evaluate relative proportions and the immediate adherence and discontinuity behavior of the three professional categories operating simultaneously within the same time frame, which may favor identification of initial trends for each service.

Regarding ethical aspects, the study complied with national data protection legislation,^21^ through the exclusive use of open-access, aggregated, and anonymized information obtained from the Brazilian Ministry of Health's monitoring dashboard website.^22^ The project was approved by the University of Brasília (UnB) Research Ethics Committee under protocol No. 6.817.982, in compliance with national health council guidelines,23., 24. ensuring confidentiality and privacy of information throughout all stages of execution.

## 3 Results

During the analyzed period, 28 services across the Federal District dispensed oral PrEP. The study population comprised 2497 individuals with a record of oral PrEP dispensation; after applying inclusion and exclusion criteria, the final sample consisted of 2317 individuals. Comparative analysis showed differences in the distribution of care according to service location (*p* < 0.001). Specialized services accounted for most of the total sample (60.5%; *n* = 1398). At this level of care, nurses (73.3%; *n* = 480) and physicians (71.2%; *n* = 880) predominated. In contrast, primary care accounted for 28.1% (*n* = 651) of total linkages. Pharmacist activity occurred predominantly in this setting, which concentrated 91.0% (*n* = 389) of this category's prescriptions. Physician and nursing care in primary care represented 8.2% (*n* = 101) and 24.6% (*n* = 161), respectively.

The administrative nature of the service also showed distinction between professional classes. The public sector encompassed the entirety of nursing (100.0%; *n* = 655) and pharmacy (100.0%; *n* = 427) activities. The medical category showed a different pattern, with private-sector engagement accounting for 63.1% (*n* = 779) of users linked to these professionals. Pharmacist prescribing is authorized only within the public network, whereas physicians may prescribe PrEP in either sector, accounting for the exclusive presence of private-sector care in the medical category. Nursing activity was likewise concentrated entirely in the public sector.

Regarding the adherence indicator (Table 1), measured by regularity of PrEP dispensations over the previous 12 months, an overall continuity rate of 79.4% (*n* = 1840) was observed. Discontinuity analysis revealed heterogeneity between groups. The medical category recorded the highest proportion of use interruption (27.6%; *n* = 341), followed by nursing (19.4%; *n* = 127). Pharmacist-followed users showed the highest percentage of prophylaxis maintenance (97.9%; *n* = 418) (Table 2).

**Table 2:** Distribution of oral PrEP encounters by service, administrative sector, and continuity, by prescribing professional category. Brasília, Federal District, Brazil, 2024–2025.

	**Total n (%)**	**Nurse n (%)**	**Pharmacist n (%)**	**Physician n (%)**	***p*-value**
**Care setting**					<0.001
Primary care	651 (28.1%)	161 (24.6%)	389 (91.0%)	101 (8.2%)	
Testing/counseling center	6 (0.2%)	3 (0.4%)	0 (0.0%)	3 (0.3%)	
Outreach (extramural)	21 (1.1%)	11 (1.7%)	0 (0.0%)	10 (0.8%)	
Specialized service	1398 (60.5%)	480 (73.3%)	38 (9.0%)	880 (71.2%)	
Private care	179 (7.4%)	0 (0.0%)	0 (0.0%)	179 (14.5%)	
Telehealth	62 (2.7%)	0 (0.0%)	0 (0.0%)	62 (5.0%)	
**Public vs. private**					<0.001
Public	1538 (66.4%)	655 (100.0%)	427 (100.0%)	456 (36.9%)	
Private	779 (33.6%)	0 (0.0%)	0 (0.0%)	779 (63.1%)	
**Dispensation in past 12 months**					<0.001
No (discontinuity)	477 (20.6%)	127 (19.4%)	9 (2.1%)	341 (27.6%)	
Yes (continuity)	1840 (79.4%)	528 (80.6%)	418 (97.9%)	894 (72.4%)	

Regarding sociodemographic characterization, a statistical association was found between professional category and race/skin color (*p* < 0.001). Medicine concentrated the highest percentage of self-declared White users (54.0%; *n* = 667). In contrast, nursing and pharmacy showed greater representation among Black and mixed-race (pardo) populations. Combined, these two groups represented 57.0% of pharmacist-attended patients (*n* = 244) and 60.2% of nursing-attended patients (*n* = 394), rates higher than the 45.0% (*n* = 556) observed in the medical category.

Regarding education level (*p* < 0.001), there was an overall predominance of users with 12 or more years of schooling (85.2%; *n* = 1973). The medical category led concentration in this stratum (90.8%; *n* = 1122). Nursing and pharmacy attended a larger share of individuals with intermediate schooling (8–11 years), at 20.3% (*n* = 133) and 13.3% (*n* = 57), respectively, compared with 7.7% (*n* = 95) recorded by medicine.

Distribution by gender identity maintained a prevalence of cisgender men above 87.5% across all groups. Furthermore, the analysis by gender showed statistical significance (*p* < 0.05). Cisgender men comprised 90.5% (*n* = 2098) of the total sample, while other gender identity profiles, such as transgender women and non-binary people, showed varying frequencies across professional categories (Table 3).

**Table 3:** Distribution of oral PrEP encounters by sociodemographic characteristics. Brasília, Federal District, Brazil, 2024–2025.

	**Total n (%)**	**Nurse n (%)**	**Pharmacist n (%)**	**Physician n (%)**	**p-value**
**Race/skin color**					<0.001
White	1091 (47.1%)	249 (38.0%)	175 (41.0%)	667 (54.0%)⁎	
Asian-descent	22 (0.9%)	6 (0.9%)	4 (1.0%)	12 (1.0%)⁎	
Mixed-race (pardo)	882 (38.1%)	282 (43.1%)⁎	180 (42.0%)	420 (34.0%)	
Black	312 (13.5%)	112 (17.1%)	64 (15.0%)⁎	136 (11.0%)	
Indigenous	10 (0.4%)	6 (0.9%)	4 (1.0%)	0 (0.0%)⁎	
**Education**					<0.001
No formal education	10 (0.4%)	5 (0.8%)	1 (0.3%)	4 (0.3%)	
1–3 years	8 (0.3%)	6 (0.9%)	0 (0.0%)	2 (0.2%)	
4–7 years	41 (1.8%)	23 (3.5%)	6 (1.4%)	12 (1.0%)	
8–11 years	285 (12.3%)	133 (20.3%)⁎	57 (13.3%)	95 (7.7%)	
12+ years	1973 (85.2%)	488 (74.5%)	363 (85.0%)	1122 (90.8%)⁎	
**Gender**					<0.05
Cisgender men	2098 (90.5%)	575 (87.8%)	383 (89.6%)	1140 (92.3%)	
Transgender men	12 (0.5%)	7 (1.1%)	0 (0.0%)	5 (0.4%)	
Cisgender women	154 (6.6%)	52 (7.9%)	35 (8.2%)	67 (5.4%)	
Transgender women	36 (1.6%)	14 (2.2%)⁎	7 (1.7%)	15 (1.2%)	
Non-binary	12 (0.5%)	3 (0.5%)	2 (0.4%)⁎	7 (0.6%)	
**Travestis**	**5 (0.3%)**	**4 (0.6%)**	**0 (0.0%)**	**1 (0.1%)**	

⁎ p < 0.05, Pearson's chi-square test (n, %). Source: study data (2025).

Vulnerability analysis identified nursing as the category with the greatest proportional reach among priority populations. This group concentrated the majority of care provided to incarcerated people (3.7%; *n* = 24), a rate higher than that recorded by pharmacists (0.2%; *n* = 1) and physicians (0.3%; *n* = 4; *p* < 0.001). A similar pattern occurred for alcohol misuse (*p* = 0.006), with a prevalence of 66.7% (*n* = 46) in nursing records versus 55.0% (*n* = 180) in medicine.

Regarding psychoactive substance use, nurses (4.3%; *n* = 28) and pharmacists (4.0%; *n* = 17) showed similar frequencies, higher than the medical category (3.0%; *n* = 37; *p* = 0.032). For sex work, nursing recorded the highest proportion (5.0%; *n* = 33), followed by pharmacists (3.7%; *n* = 16) and physicians (2.2%; *n* = 27; *p* = 0.006). The homelessness variable showed no statistical difference between professional categories (*p* = 0.218) (Table 4).

**Table 4:** Programmatic and social vulnerability profile of patients by professional category (nurse, pharmacist, physician). Brasília, Federal District, Brazil, 2024–2025.

**Variable**	**Total n (%)**	**Nurse n (%)**	**Pharmacist n (%)**	**Physician n (%)**	**p-value**
**Homelessness**					0.218
Yes	5 (0.2%)	3 (0.5%)	0 (0.0%)	2 (0.2%)	
No	2312 (99.8%)	652 (99.5%)	427 (100.0%)	1233 (99.8%)	
**Incarceration**					<0.001
Yes	29 (1.3%)	24 (3.7%)*	1 (0.2%)	4 (0.3%)	
**No**	**2288 (98.7%)**	**631 (96.3%)**	**426 (99.8%)**	**1231 (99.7%)**	
Alcohol misuse†					0.006
Yes	226 (57.1%)	46 (66.7%)*	–	180 (55.0%)	
No	170 (42.9%)	23 (33.3%)	–	147 (45.0%)	
**Psychoactive substance use**					0.032
Yes	82 (3.5%)	28 (4.3%)*	17 (4.0%)	37 (3.0%)	
No	2235 (96.5%)	627 (95.7%)	410 (96.0%)	1198 (97.0%)	
**Sex work**					0.006
Yes	76 (3.3%)	33 (5.0%)*	16 (3.7%)	27 (2.2%)	
No	2241 (96.7%)	622 (95.1%)	411 (96.3%)	1208 (97.8%)	

*p < 0.05, Pearson's chi-square test (n, %). † Alcohol misuse was not assessed among pharmacist-attended patients during the study period; the total and p-value for this variable are based on nurse- and physician-attended patients only (n = 396). Source: study data (2025).

Analysis of adherence to the prophylaxis pickup schedule showed statistically significant disparities between professional categories (*p* < 0.001). The highest rate of timeliness at each new pickup was observed among pharmacist-followed users (87.6%; *n* = 418), followed by those assisted by nursing (83.5%; *n* = 652), while the medical category recorded the lowest adherence rate to the resupply flow (73.4%; *n* = 1247).

To verify whether the association between pharmacist and nursing care and greater user retention held independently of vulnerability profile, multivariate logistic regression was performed (Table 5). The analysis indicated that the pharmacist care model was an independent predictor of continuity of PrEP use, conferring 4.25-fold greater odds of retention (aOR = 4.25; 95%CI: 2.10–8.50; *p* < 0.001) compared with physician-led follow-up. Nursing activity also showed a positive and significant association with prophylaxis maintenance (aOR = 1.65; 95%CI: 1.30–2.10; *p* = 0.002), even after adjustment for vulnerability factors, such as incarceration and alcohol misuse, which correlated inversely with continuity of prophylaxis use.

**Table 5:** Adjusted multivariate logistic regression for continuity of oral PrEP use, by professional category and vulnerability factors. Brasília, Federal District, Brazil, 2024–2025.

	**aOR**	**95% CI**	**p-value**
**Professional category (Ref: Physician)**			
Pharmacist	4.25	2.10–8.50	<0.001
Nurse	1.65	1.30–2.10	0.002
Incarcerated (Yes)	0.45	0.25–0.80	0.007
Alcohol misuse (Yes)	0.6	0.45–0.80	<0.001

p < 0.05. Source: study data (2025).

## 4 Discussion

This study aimed to analyze the association between prescriber professional category and continuity of oral PrEP use, examining the contribution of nursing and pharmacy to strengthening access and retention among vulnerable populations within a universal public health system. Findings indicated that expansion of PrEP prescribing beyond medicine, incorporating pharmacists into prescribing already performed by nurses, was associated with an observable reorganization of the care network, particularly in primary care and the public sector.^10^, 15., 25. This configuration converged with national and international combination-prevention guidelines, which advocate decentralization and task-sharing as central strategies for expanding access and bringing prevention technologies closer to populations most vulnerable to HIV.^7^, 8.

The asymmetric distribution between the public and private sectors highlighted the segmentation in the organization of PrEP care within the Federal District. The data indicated that access pathways differed according to the type of healthcare provider involved, with physician-led PrEP follow-up occurring predominantly in the private sector, while medication provision remained linked to the public health system through SUS pharmacies. In contrast, pharmacist- and nurse-led prescribing were regulated within the public network and provided free of charge. This configuration positioned multiprofessional practice as a structural component of equitable PrEP delivery rather than a complementary approach.^18^, ^19^, ^20^

The greater involvement of nurses and pharmacists among Black and mixed-race users reinforced this reading. National data show that Black people remain overrepresented among new HIV diagnoses and underrepresented among prophylaxis users.1., 4 The cross-sectional design did not allow establishing a causal relationship between prescribing category and user profile, but the data suggest that these categories occupied a strategic position in reducing racial inequities in PrEP access.^20^, ^26^, ^27^

The proximity of nursing to priority populations has been documented in studies identifying reception practices, qualified listening, and territorial engagement as explanatory factors.^20^, ^28^, ^29^ The literature on pharmaceutical care in HIV similarly highlights these professionals' capacity to establish continued therapeutic relationships, review treatment regimens, and support adherence through structured follow-up, often with more time dedicated per consultation.^30^, 31.

The pattern of greater nursing involvement with incarcerated people, people who use psychoactive substances, and sex workers confirmed this category's role at the interface with groups that accumulate social and programmatic exclusions.^20^, ^32^ Institutional stigma and criminalization of substance use hinder continuous access to PrEP, requiring approaches based on harm reduction and intersectorality.^5^, ^33^

Pharmacist involvement with priority populations, although lower than that of nursing, exceeded that of medicine on the variables of greatest vulnerability. This result should not be interpreted as unsatisfactory performance, but as a starting point for an engagement still under consolidation. International experiences have reported high acceptability of pharmacist PrEP prescribing, with positive user perceptions regarding reduced moral judgment and favorable retention rates compared with physician-only models.9., 15., 34 A pharmacist-led PrEP service in Nova Scotia, Canada, delivered through community pharmacies, similarly reported high patient acceptance and few privacy or stigma-related concerns.^34^ International HIV bodies have incorporated pharmacist-led delivery into differentiated service delivery guidance for PrEP.^15^

The continuity rate among pharmacist-followed users was the standout finding but requires cautious interpretation. This result aligned with evidence associating structured pharmacist follow-up with better adherence in conditions such as hypertension, diabetes, and HIV.^30^, 31. However, unlike physicians and nurses, who were already prescribing PrEP before the analyzed period, pharmacist prescribing was introduced only in 2024 within the Federal District, which structurally reduced this category's time of exposure to discontinuity risk. Ignoring this temporal asymmetry would be equivalent to comparing populations with different observation windows, compromising the validity of inferences.^35^

Longitudinal studies indicate that PrEP discontinuation tends to increase with follow-up time, influenced by factors such as changing risk perception, adverse events, and logistical barriers.^3^, ^36^ Consolidating evidence on pharmacist performance in retention will require analyses with longer follow-up and control for confounding factors such as self-reported risk profile and service-use patterns.^17^

The greater discontinuity observed among medically followed users was associated with this group's predominance in the private sector and the possibly higher income and education profile of the users seen. Part of this discontinuity may not represent actual abandonment of prophylaxis: users with greater autonomy tend to demand intermittent PrEP regimens, which is reflected in less regular dispensation patterns without necessarily indicating clinical interruption. This distinction between programmatic discontinuity and actual interruption reinforces the need for triangulation with qualitative and behavioral data for adequate interpretation of monitoring indicators.^36^, ^37^

The underrepresentation of transgender people, *travestis*, and other gender-dissident identities, despite formal recognition of these populations as priorities, was a finding that transcended the comparative scope of the study. Experiences of stigma, discrimination, and institutional violence have been identified in the literature as decisive barriers to access and retention in prevention technologies. Expansion of multiprofessional prescribing alone did not resolve this structural barrier.^4^, ^20^, ^38^

The results reinforced the argument that mere expansion of PrEP supply is not sufficient to guarantee full equity.16., 33 Concentration of pharmacist prescriptions in primary care indicated that this category operated under a logic distinct from the others, structurally more aligned with the decentralization advocated by national and international guidelines.^10^, 25., 28 More successful interventions, according to the literature, combine decentralization with community-based approaches, integration with mental health services, and harm-reduction mechanisms.15., 39

Continuity of care assessed by dispensation regularity converged with international evidence on the pharmacist's role in PrEP program engagement. McElyea et al.^14^ found that embedding a clinical pharmacist within a model targeting people experiencing homelessness resulted in greater outreach and user linkage. Greenwell et al.^40^ found less loss to follow-up in the group followed by pharmacists via telehealth compared with the in-person physician model, even though clinical adherence was similar between the two models. These findings reinforced that the pharmacist care model did not compromise clinical quality and favored longitudinal engagement with the service.

As a strength, the study offered a comparative picture of multiprofessional PrEP prescribing within a universal public health system during a period of policy expansion.^5^, ^32^ Among the limitations, the cross-sectional design does not allow establishing causal relationships between prescriber category and the outcomes analyzed. We also highlight the use of secondary, aggregated data, which did not allow access to information on individual behaviors or subjective reasons for discontinuation. Because pharmacist prescribing authorization is restricted to the public health network, professional category and care sector are structurally linked in this setting, limiting the extent to which a professional-category effect can be statistically disentangled from a care-setting effect. Additionally, although the analyzed period (October 2024–December 2025) was defined to begin only after pharmacist prescribing became available, avoiding a comparison against categories with decades of prior practice, the more recent and still-expanding nature of pharmacist prescribing within this window likely resulted in a shorter average follow-up time for this category, and this structurally shorter time of insertion within the monitoring system limits direct longitudinal comparisons with the other categories. Because users followed for less than 12 months were evaluated by the timely-dispensation criterion rather than the cumulative 12-month retention criterion, a category with proportionally more recently enrolled users, such as pharmacists, was more likely to be assessed under the more lenient criterion, which may have inflated its apparent continuity rate relative to physicians and nurses.^20^, ^34^, ^36^, ^41^

The results opened perspectives for future studies. Cohorts with extended follow-up would allow assessment of the sustainability of the observed continuity rates.^3^, ^17^ Mixed-methods research could explore subjective reasons for discontinuation according to prescribing category. Replicating the design in other jurisdictions would contribute to assessing the generalizability of findings and identifying contextual factors that modulate each category's reach among vulnerable populations.^28^ Specific investigations into access for transgender people and *travestis* according to care model would be relevant for guiding differentiated reception and retention strategies.^4^, ^6^

The results supported continuity of task-sharing and decentralization policies, aligned with guidelines that place equity at the center of strategies toward eliminating AIDS as a public health problem by 2030, provided they are accompanied by longitudinal monitoring, continuing education, and discrimination-free reception mechanisms.1., 8., 25., 30, 38

## 5 Conclusion

Findings indicated that integration of pharmacist prescribing and the role of nursing within the public network of the Federal District were associated with broader reach among historically vulnerable populations and higher rates of initial prophylaxis continuity compared with the physician-centered model, with the concentration of pharmacist prescriptions in primary care signaling greater alignment of this category with the decentralization advocated by national and international combination-prevention guidelines. Accordingly, the results support continuity of task-sharing and prescribing decentralization policies as strategies to strengthen access and retention of vulnerable populations in PrEP within the public health system, signaling that multiprofessional prescribing can contribute to reducing inequities in access to combination prevention technologies and to bringing the national HIV response closer to AIDS elimination goals as a public health problem.

## Declaration of generative AI and AI-assisted technologies IN the manuscript preparation process

During the preparation of this work, the authors used Claude (Anthropic) to assist with translation into English, reference formatting and numbering, and restructuring of the manuscript to comply with journal submission guidelines. The study design, data analysis, results, and interpretation are entirely the authors' own. After using this tool, the authors reviewed and edited the content as needed and take full responsibility for the content of the published article.

## CRediT authorship contribution statement

**Francisco Álisson Paula de França:** Writing – review & editing, Writing – original draft, Software, Methodology, Investigation, Data curation, Conceptualization. **Ana Luiza Alves dos Santos:** Software, Investigation, Formal analysis, Data curation. **Gabriel Gonçalves Okamoto:** Validation, Supervision. **Maria Christina dos Santos Verdam:** Visualization, Validation, Methodology. **Débora Santos Lula Barros:** Visualization, Supervision, Software. **Noemia Urruth Leão Tavares:** Validation, Supervision, Methodology. **Rodrigo Fonseca Lima:** Visualization, Validation, Project administration, Methodology. **Rafael Santos Santana:** Validation, Supervision.

## Funding

This study did not receive funding from public, commercial, or not-for-profit funding agencies.

## Declaration of competing interest

The authors declare that they have no known competing financial interests or personal relationships that could have appeared to influence the work reported in this paper.

## Data Availability

The aggregated, anonymized data used in this study were obtained from the Brazilian Ministry of Health's national PrEP monitoring dashboard, which is openly accessible online. Additional extractions stratified by prescribing professional category, not openly available on the dashboard, can be obtained from the corresponding author upon reasonable, justified request and subject to applicable data protection requirements.
